# Molecular and morphological characterisation of larvae of the genus *Diamesa* Meigen, 1835 (Diptera: Chironomidae) in Alpine streams (Ötztal Alps, Austria)

**DOI:** 10.1371/journal.pone.0298367

**Published:** 2024-02-15

**Authors:** Martin Dvorak, Isabel L. Dittmann, Veronika Pedrini-Martha, Ladislav Hamerlík, Peter Bitušík, Evzen Stuchlik, Daniel Vondrák, Leopold Füreder, Reinhard Lackner

**Affiliations:** 1 Institute of Zoology, University of Innsbruck, Innsbruck, Austria; 2 Faculty of Natural Sciences, Matej Bel University, Banská Bystrica, Slovakia; 3 Institute of Zoology, Slovak Academy of Sciences, Bratislava, Slovakia; 4 Institute of Hydrobiology, Biology Centre, Czech Academy of Sciences, České Budějovice, Czech Republic; 5 Institute for Environmental Studies, Faculty of Science, Charles University, Prague, Czech Republic; 6 Institute of Ecology, University of Innsbruck, Innsbruck, Austria; Universitat de Barcelona, SPAIN

## Abstract

*Diamesa* species (Diptera, Chironomidae) are widely distributed in freshwater ecosystems, and their life cycles are closely linked to environmental variables such as temperature, water quality, and sediment composition. Their sensitivity to environmental changes, particularly in response to pollution and habitat alterations, makes them valuable indicators of ecosystem health. The challenges associated with the morphological identification of larvae invoke the use of DNA barcoding for species determination. The mitochondrial cytochrome oxidase subunit I (COI) gene is regularly used for species identification but faces limitations, such as similar sequences in closely related species. To overcome this, we explored the use of the internal transcribed spacers (ITS) region in addition to COI for *Diamesa* larvae identification. Therefore, this study employs a combination of molecular markers alongside traditional morphological identification to enhance species discrimination. In total, 129 specimens were analysed, of which 101 were sampled from a glacier-fed stream in Rotmoostal, and the remaining 28 from spring-fed streams in the neighbouring valleys of Königstal and Timmelstal. This study reveals the inadequacy of utilizing single COI or ITS genes for comprehensive species differentiation within the genus Diamesa. However, the combined application of COI and ITS markers significantly enhances species identification resolution, surpassing the limitations faced by traditional taxonomists. Notably, this is evident in cases involving morphologically indistinguishable species, such as *Diamesa latitarsis* and *Diamesa modesta*. It highlights the potential of employing a multi-marker approach for more accurate and reliable Diamesa species identification. This method can be a powerful tool for identifying *Diamesa* species, shedding light on their remarkable adaptations to extreme environments and the impacts of environmental changes on their populations.

## Introduction

The most striking effects of global climate change in the Alps include retreating glaciers [1] and an increasing temperature in mountain streams [2]. This process has far-reaching consequences, such as a shift in composition of the benthic fauna, where cold-adapted species are replaced by species from lower altitudes [3]. Tiny flies of the Chironomidae family (Diptera, Nematocera), informally known as chironomids, are one of the typical groups of aquatic insects that inhabit cold streams, including glacier-fed streams. Despite harsh conditions, cold streams in the European Alps host a dense but highly specialized invertebrate fauna, where chironomids are often the only group present [4, 5]. However, this only applies to specific taxa, as chironomids inhabit water ecosystems with a wide range of temperatures [6, 7].

Larvae of the Diamesinae subfamily, especially those of the *Diamesa* genus, are the most abundant chironomids of glacier-fed streams (kryal), even located by the glacier terminus; however, they can also dominate in cold streams not affected by meltwater (krenal, rhitral) [3, 8, 9]. Larval *Diamesa* are generally adapted to cold and well-oxygenated waters, springs, and occasionally to shallow stagnant waters and madicolous sites [10, 11]. Some *Diamesa* species live at high altitudes up to 5,600 m a.s.l. [12] and references therein and can complete their life cycles even when water temperature do not exceed 2°C [13, 14]. It is obvious that *Diamesa* species could play a key role in the assessment of changes in high-altitude streams and springs under climatic change [15]. However, effective monitoring is hampered by objective difficulties with the reliable identification of larval *Diamesa*.

In the Palaearctic Region, the genus comprises more than 100 species [16], 38 of which are known from Europe [17]. Reliable identification of the species is based on adult males [18] and to some extent on the pupae and pupal exuviae [19]. Even though modern identification keys are available for *Diamesa* larvae (e.g., [20, 21]), larval identification is a time-consuming and difficult process even for experienced taxonomists. Moreover, key structures, such as mentum and mandibles, may be worn or damaged, making identification even more difficult. In addition, some *Diamesa* species are indistinguishable as larvae. Thus, using up-to-date molecular methods along with morphological diagnosis is crucial for distinguishing similar species [22–24].

This study aims to identify *Diamesa* species from brooks in the Ötztal Alps, Austria, and compare the morphological identification of larvae with the identification based on molecular markers using DNA barcoding methods. The success of DNA barcoding relies on the availability of reference sequences for comparison. The COI gene has been widely adopted as a universal DNA barcode marker across various taxa [25–27]. The COI sequences are more commonly available in public databases, making it easier to match and identify unknown specimens.

The limitations of using COI alone for barcoding, such as similarities in sequences among closely related species and intraspecific variations [28–31], prompted consideration of suitable alternatives. Several studies have highlighted the potential of the internal transcribed spacer (ITS) region within ribosomal DNA for species identifications, including animals, plants, and fungi [32–35]. The ITS region, located in the nuclear genome, exhibits lower intraspecific variability than COI while maintaining adequate interspecific variability, making it a perfect marker for species delineation [36]. Despite the usefulness of the nuclear ITS region in increasing the resolution of species identification, there are reasons why using only ITS might not be the optimal approach [37]. The molecular evolution rates of mitochondrial and nuclear genes can differ. Mitochondrial genes, like COI, often evolve faster than nuclear genes [38]. This faster evolution can be advantageous for distinguishing closely related species. Depending solely on the slower-evolving ITS may lead to insufficient divergence for accurate species discrimination in certain cases. Relying solely on ITS might also result in a lack of consistency and comparability with existing DNA barcode databases. The combination of COI and ITS proved to be the most effective approach, allowing accurate sorting of species into distinct groups [36, 37]. Combining both mitochondrial and nuclear markers provides complementary information [37]. Mitochondrial markers are maternally inherited and can provide insights into historical relationships, while nuclear markers offer information on recent evolutionary events [39]. The combination of both markers enhances the overall resolution and accuracy of species delineation [36, 37].

One potential application of DNA barcoding for *Diamesa* species identification is in the monitoring of current environmental changes. As mentioned earlier, global warming and related environmental changes are causing the disappearance of glaciers in the Alps and an increase in the water temperature of mountain streams. We assume that chironomids of the *Diamesa* genus have a great bioindication potential and can be used to assess future changes in the quality of these vulnerable ecosystems.

## Materials and methods

### Sampling

**Fig 1:**
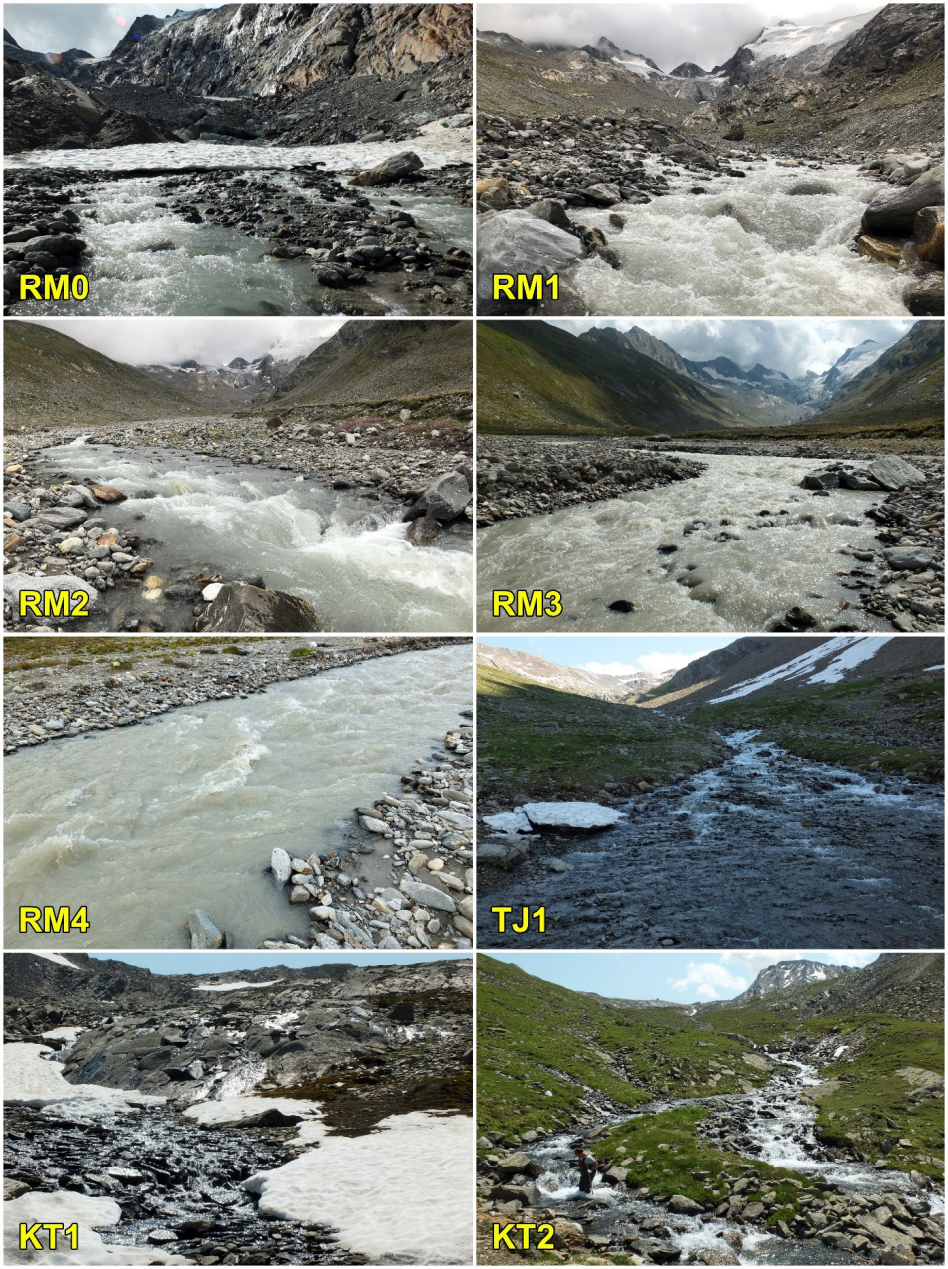
Sampling sites in the Ötztal Alps. Sampling on August 22, 2020 (RM3, RM4), August 23, 2020 (RM0, RM1, RM2), and July 20, 2021 (TJ1, KT1, KT2). For sapling site codes see Table 1; Photos: D. Vondrák.

Sampling sites are situated near Obergurgl (community of Sölden, Tyrol, Austria) in three neighbouring valleys–Rotmoostal, Königstal, and Timmelstal. The focus was on Rotmoostal, where a stream (Rotmoosache) is fed by meltwater from the Rotmoosferner and the Wasserfallferner. Rotmoos valley is part of the LTER site Obergurgl belonging to the LTSER platform Tyrolean Alps and to the national and international long-term ecological research networks (LTER-Austria, LTER Europe, and ILTER). In that stream, we expected the harshest conditions out of the three sampled streams and the presence of the *Diamesa*-dominated chironomid fauna. The other alpine streams in the Königstal and Timmelstal valleys (Königsbach and Timmelsbach) are spring-fed streams with no glaciers in their catchments. The sampling sites (five in the Rotmoostal, two in the Königstal, and one in the Timmelstal) were selected to cover a gradient of harshness (see [40], Fig 1, Table 1 for details). All sampling sites are situated above the treeline. The local bedrock is composed of granitic rocks with marble layers, feldspar, and mica schists. Chironomid larvae were sampled using a kick-sampling net (frame size of 25 × 17 cm, mesh size of 500 μm, sampling time approximately 5 minutes), stored in a thermos flask, and immediately transferred to a laboratory in the nearby University Center Obergurgl, Austria, and sorted under a dissecting microscope. Chironomids of the genus *Diamesa* were subsequently treated in a specific way (see below).

**Table 1:** Basic parameters of the sampling sites. Abbreviations: GF–glacier-fed, NG–non-glacial.

Valley		Rotmoostal					Königstal		Timmelstal
Stream		Rotmoosache					Königsbach		Timmelsbach
Site code		RM0	RM1	RM2	RM3	RM4	KT1	KT2	TJ1
Stream type		GF	GF	GF	GF	GF	NG	NG	NG
Sampling date		23.8.2020 21.7.2021	23.8.2020	23.8.2020	22.8.2020	22.8.2020 21.7.2021	20.7.2021	20.7.2021	19.7.2021
Coordinates	N	46°49.394’	46°49.705’	46°50.162’	46°50.391’	46°50.727’	46°52.056’	46°52.467’	46°54.044’
	E	11°2.670’	11°2.495’	11°2.176’	11°1.865’	11°1.141’	11°4.493’	11°4.073’	11°5.528’
Altitude (m)		2515	2385	2335	2285	2260	2670	2450	2405
Exposition of the valley		NW	NW	NW	NW	NW	NW	NW	N
Distance from glacier (km)		0.02	0.7	1.7	2.2	3.5	-	-	-
Bottom substrate		boulders, cobble, pebble, sand, glacial flour	boulders, cobble, pebble, sand, glacial flour	boulders, cobble, pebble, sand, glacial flour	boulders, cobble, pebble, sand, glacial flour	boulders, cobble, pebble, sand, glacial flour	rock, boulders, cobble, pebble, sand, aquatic moss	rock, boulders, cobble, pebble, sand	rock, boulders, cobble, pebble, sand

### Morphological identification

**Fig 2:**
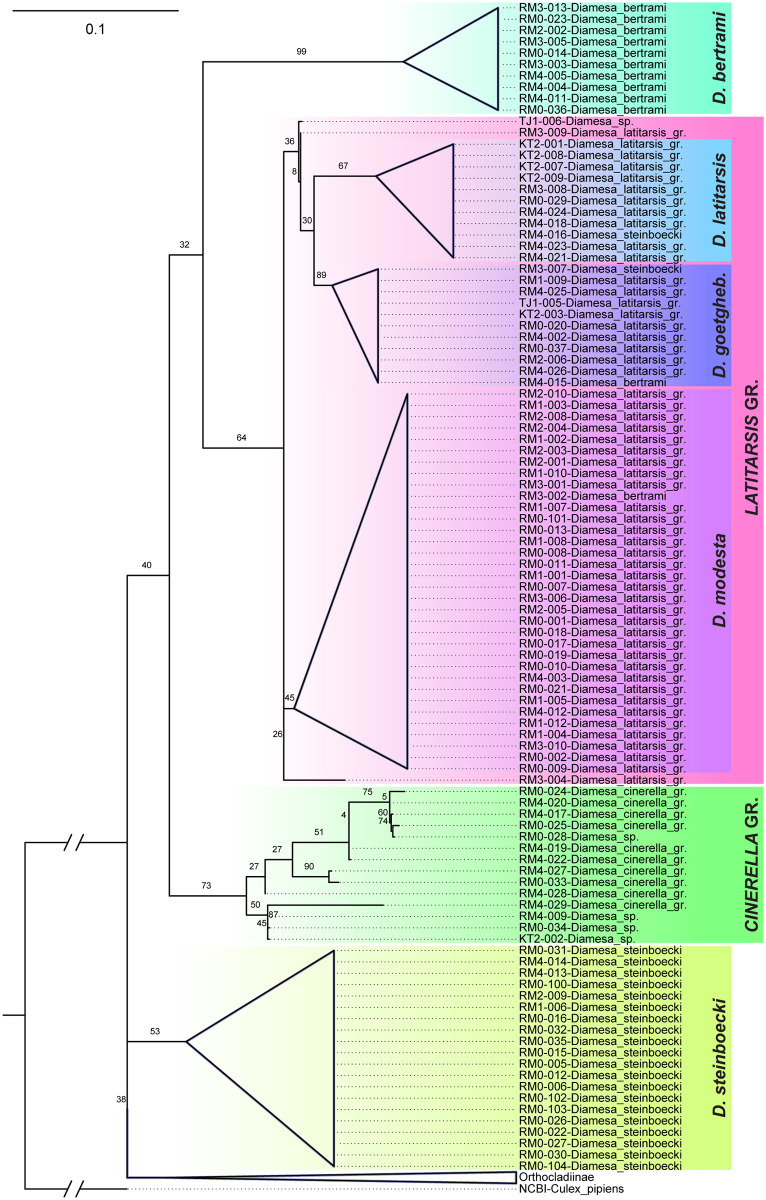
Phylogenetic tree showing species delimitation analysis based on the combination of COI marker genes and internal transcribed spacer (ITS) sequences. The first three letters in each individual’s code represent the sampling site code (see Table 1). The species names after the sample ID refer to the morphological identification, while colour coding for species is based on molecular assignments.

Each larva was dissected in a drop of distilled water to prevent drying and collapsing body structures. The head and the posterior part of the abdomen of each specimen were embedded in the Euparal microscopy mountant (Carl Roth GmbH + Co. KG, Karlsruhe, Germany) on microscope slides for further taxonomic identification. The posterior part of the abdomen always included posterior parapodes, anal tubules, and anal setae, and the head was oriented by the ventral side up to show important morphological characters. Taxonomic identification followed Rossaro and Lencioni [20, 21]. Results of morphological identification are shown after sample ID in Fig 2. If individual species were morphologically indistinguishable, we tried to assign particular samples to respective clades and indicated that with the group abbreviation (gr.) in the sample name.

### DNA extraction, PCR amplification and sequencing

A small part of the remaining body (up to 1 mm) was used for DNA extraction by the prepGem Universal kit (MicroGem; Southhampton, UK). Briefly, 44 μl of dd H_2_O DEPC, 5 μl of 10x blue buffer (prepGem Universal kit), and 1 μl of prepGEM were added to the sample. After 15 min at 75°C, the sample was heated up for 2 min to 95°C. After cooling, samples were stored at -20°C and were ready to be used for DNA barcoding.

PCR was performed with Advantage 2 Polymerase Mix (Takara, Japan). Primers sequences were outsourced from Folmer *et al*. [41] (LCO1490: 5’-ggtcaacaaatcataaagatattgg-3’, HC02198: 5’-taaacttcagggtgaccaaaaaatca-3’) with an expected product length of 658 base pairs. Briefly, a denaturation cycle of 95°C for 8 min was followed by 33 cycles with 95°C for 30 s, 57°C for 30 s, and 68°C for 1 min, with a final 10 min extension step at 70°C. PCR products for sequencing with Microsynth (Switzerland) were cleaned by QIAquick PCR Purification Kit (Qiagen, Germany). For quality control, amplified fragments were separated by size through electrophoresis on a 1.5% agarose gel in TEA buffer (40 mM Tris, 20 mM acetic acid, and 1 mM EDTA).

To improve resolution, we performed additional identification based on a section of nuclear ribosomal ITS. Primers were outsourced from Newburn & Krane [42] (18S: 5’-gatgttctgggcggcacgcg-3’, 28S: 5’-ttggtttcttttcctcccct-3’). PCR reactions of 25 μl were performed as follows: a denaturation cycle of 95°C for 8 min was followed by 30 cycles with 95°C for 30 s, 62.5°C for 30 s and 68°C for 1 min, with a final 10 min extension step at 70°C.

### Phylogenetic analyses

Sequences for each gene were separately aligned with MAFFT G-INS-i v7.490 [43]. They were manually trimmed and concatenated to form a supermatrix (COI 1-453bp + ITS 454-3088bp). For phylogenetic reconstruction, we pursued maximum likelihood reconstructions using RAxML v8.2.10 [44]. The best model (GTR+G+I, same for both) was determined for individual alignments by MEGA software v11.0.13 [45]. The best ML tree was selected out of 1000 tree searches and 1000 separate bootstrap replicates were performed. Bootstrap support was mapped for each supermatrix onto the best ML tree. This was done both for the concatenated supermatrix (Fig 2) and for individual genes separately (S1 and S2 Figs). In the case of COI, we included known chironomid sequences from the database (S3 Table) to enhance the identification of species by clustering these known sequences with the clusters formed by our samples. This was crucial for the identification of morphologically indistinguishable larval samples, such as *Diamesa modesta* and *Diamesa latitarsis*. There were no records of *Diamesa* ITS sequences in GenBank at the time of this study (2023). Phylogenetic trees were visualised in Figtree v1.4.4 (http://tree.bio.ed.ac.uk/software/figtree/) and redesigned in Adobe Illustrator CC (2014). The analysed sequences are available in the GenBank database under respective accession numbers (S2 Table). Further datasets from this study are available from the corresponding author upon request.

## Results

In total, 129 specimens were analysed, with 101 sampled from the glacier-fed stream of Rotmoostal and the remaining 28 from the spring-fed streams in neighbouring valleys of Königstal and Timmelstal. This study focused on the subfamily Diamesinae, particularly the genus *Diamesa* (in total 104 specimens). However, several other taxa from the subfamilies Orthocladiinae and Chironominae were also analysed as outliers or accidentally in the case of small and dark Orthocladiinae taxa that resembled *Diamesa* larvae. Among the chironomid specimens collected from the study area, three *Diamesa* species were identified to occur only in the glacial-fed stream of Rotmoostal and were absent in the samples from the spring-fed streams of Königstal and Timmelstal (KT1, KT2, and TJ1). These exclusively glacial-fed stream species were *Diamesa bertrami* Edwards, 1935, *D*. *modesta* Serra-Tosio, 1967, and *D*. *steinboecki* Goetghebuer, 1933 (Table 2). Regarding the spring-fed streams, they were dominated by the subfamily Orthocladiinae and Chironominae. Particularly at the sampling site KT1, *Diamesa* species were completely absent in our sample set (Table 2).

**Table 2:** *Diamesa* occurrence at the sampling sites according to the phylogenetic tree (see Fig 2).

Valley	Rotmoostal					Königstal		Timmelstal
Stream	Rotmoosache					Königsbach		Timmelsbach
Site code	RM0	RM1	RM2	RM3	RM4	KT1	KT2	TJ1
*Diamesa bertrami*	x		x	x	x			
*Diamesa latitarsis*	x			x	x		x	
*Diamesa goetghebueri*	x	x	x	x	x		x	x
*Diamesa modesta*	x	x	x	x	x			
*Diamesa cinerella* group	x				x		x	
*Diamesa steinboecki*	x	x	x		x			

The phylogenetic tree species delimitation analysis, illustrated in S1 and S2 Figs uses cytochrome oxidase subunit 1 (COI) marker gene sequences and internal transcribed spacer 1 and 2 (ITS) marker sequences. The COI tree (S1 Fig), incorporating known GenBank sequences as a reference, facilitated the identification of distinct species clusters. Notably, despite the morphologically indistinguishable species of *Diamesa modesta* and *Diamesa latitarsis*, these sequences formed separate clusters, with *D*. *modesta* aligning well with the known GenBank sequence (S3 Table). The second cluster did not align with the *Diamesa latitarsis* sequence from the GenBank (S3 Table); however, it was distinct from the cluster confidently identified as *Diamesa modesta*. Given the certainty of our morphological identification of larval samples as either *modesta* or *latitarsis*, we confidently classify the second cluster as *Diamesa latitarsis*. Discrepancies between the GenBank sequence and our sequences may stem from limitations inherent in the COI gene described earlier.

Nevertheless, the individual COI and ITS phylogenetic trees revealed a lack of adequate species resolution based on morphological analysis. To address this limitation and distinguish between *Diamesa* species, we employed concatenated sequences.

In the resulting phylogenetic tree (Fig 2), two clades of the *Diamesa* cluster were polyphyletic with a clade of Orthocladiinae. The main clade comprises *Diamesa steinboecki*, *Diamesa bertrami*, the members of the *Diamesa cinerella* group (likely *D*. *cinerella* Meigen, 1835 and *D*. *starmachi* Kownacki & Kownacka, 1970), and the so-called *Diamesa latitarsis* group (mainly *D*. *modesta*, *D*. *latitarsis* Goetghebuer, 1921, *D*. *goetghebueri* Pagast, 1947). The tree topology shows that the *D*. *cinerella* group builds a sister group to *D*. *steinboecki*, *D*. *bertrami*, as well as to the *D*. *latitarsis* group. In our tree, the *D*. *latitarsis* group mainly comprises sequences of *D*. *goetghebueri*, *D*. *modesta* and *D*. *latitarsis*. Besides a few exceptions, the representatives of the corresponding species appear monophyletic, forming three clades (Fig 2). The first branching is *D*. *modesta; D*. *goetghebueri* and *D*. *latitarsis* appear as sister clades. Only three sequences (RM3-004-*Diamesa_latitarsis* gr., TJ1-006-*Diamesa*_sp., and RM3-009-*Diamesa_latitarsis*_gr.; see Fig 2) of the *D*. *latitarsis* group cluster separately and do not associate with any of the three main clades formed by *D*. *goetghebueri*, *D*. *modesta*, or *D*. *latitarsis*.

## Discussion

In recent years, the use of DNA barcoding in species identification has gained increasing popularity in many fields of biology. DNA barcoding is a technique that involves the use of a short DNA sequence to identify species. The DNA sequence used for barcoding is usually a fragment of a specific gene, such as the mitochondrial cytochrome oxidase subunit I (COI) gene. The COI gene is highly conserved within species but highly variable between species, making it an excellent tool for species identification. However, there are some limitations to the use of the COI gene as a barcode marker for species determination [22]. One of the limitations is that some closely related species may have very similar COI gene sequences, making it difficult to distinguish between them [22, 46]. In addition, some species may have intraspecific variations in their COI gene sequence, leading to potential misidentification [47]. Although established methods of DNA barcoding for chironomid species utilize fragments of the mitochondrial COI, it doesn’t provide sufficient resolution to distinguish all *Diamesa* species (S1 Fig).

To overcome these limitations and to increase the resolution of barcoding, we have been exploring the use of additional genetic markers for DNA barcoding. One such marker is the ITS region, which is located between the 18S and 26S rRNA genes in the nuclear genome. The ITS region has been found to have higher variability than the COI gene, making it a potentially useful marker for species delineation. The ITS is generally used for barcoding studies with animals and plants [32, 33, 35]. While the ITS alone improved the resolution of studied species, it exhibits some level of conservation within closely related species. In our case, this mainly concerns the *Diamesa latitarsis* group, where the individual species within this taxonomic cluster exhibited insufficient separation into distinct clusters (S2 Fig). For other clades, *Diamesa bertrami*, *steinboecki*, *cinerella* gr. and *Orthocladine* sp. separation was sufficient (S2 Fig). The most effective approach was a combination of COI and ITS together and a running analysis of the combined sequence of both. In *Diamesa* larvae, the combination of both markers allows for more accurate species delineation. This method enabled us to sort all analysed species into species groups. Our findings were compared with already published sequences from the NCBI database and the results of our taxonomical identification based on morphological features.

Identifying *Diamesa* larvae to species level is a difficult task due to the heavily sclerotized, frequently dark head, often worn mouthparts (mentum and mandibles), extreme morphological similarity of *Diamesa* species, and high variability of morphological features even within a species. This similarity is particularly evident among larvae of closely related species (i.e., members of a species group), which may share the same habitats and have similar ecological niches. The challenge of identifying *Diamesa* larvae to the species level is compounded by the fact that only larvae of about one-third of the described species are known [11], and there are quite few identification keys dealing with them (e.g., [20, 21, 48–51]); the precise identification of the genus is mainly based on the morphology of the adult males ([52]). Nevertheless, it is difficult to identify the larvae in the absence of adult males, and alternatively pupal exuviae or pharate adults. Additionally, the morphology of *Diamesa* larvae is subject to a high degree of plasticity in response to environmental factors, such as temperature, pH, and nutrient availability. This variability can make it difficult to distinguish between different species, as their morphological features may overlap or change in response to different environmental conditions [46]. Our tree shows that using the molecular marker gene COI in combination with ITS enables species identification even when traditional taxonomy reaches its limits.

Such an example is the differentiation between members of the so-called *Diamesa latitarsis* group [22, 46]. Besides the eponym species, *D*. *latitarsis*, this group includes six further species occurring in the Alps: *D*. *goetghebueri*, *D*. *laticauda* Serra-Tosio, 1964, *D*. *lindrothi* Goetghebuer, 1931, *D*. *martae* Kownacki & Kownacka, 1980 and *D*. *modesta*. These species were grouped based on the morphological features of the adult males [10, 53]. Montagna *et al*. [22] and Lencioni *et al*. [46] showed that this similarity of features likely has a shared common evolutionary origin, as this group appears monophyletic also in molecular phylogenetic analyses. In our study (Fig 2), the *D*. *latitarsis* group is mainly represented by *D*. *goetghebueri*, *D*. *modesta* and *D*. *latitarsis* which were distinguished based on the molecular data and form the three distinct monophyletic clades. At present, *D*. *modesta* is indistinguishable from *D*. *latitarsis* using morphological characters, and *D*. *goetghebueri* can only be distinguished in some larvae (see [20, 21]). We combined the results of our molecular analysis with COI alignment to known sequences of the NCBI database (S3 Table). With the help of our phylogenetic tree majority of these cases of potential misidentifications are easy to recognize and assign to the correct species (Fig 2). However, more difficult is the interpretation of the sequences clustering besides the main clades (RM3-004-*Diamesa_latitarsis*_gr., TJ1-006-*Diamesa*_sp., RM3-009-*Diamesa_latitarsis*_gr.; see Fig 2). Just with morphological characters and without further molecular references we were not able to identify the samples to the species level. As they do not cluster within the main clades in the tree, it might be possible that each of these sequences represents one of the remaining alpine species of the *D*. *latitarsis* group, i.e., *D*. *laticauda*, *D*. *lindrothi* or *D*. *martae* (larvae of the last two mentioned are unknown). However, we would need more data to clarify the phylogenetic affiliation of these sequences. However, the species mentioned above are not listed in the identification keys we used [20, 21] and consequently were not considered during morphological identification.

A similar problem arises in the clade *D*. *cinerella* group. In our phylogenetic tree, the *D*. *cinerella* group cluster does not seem monophyletic as *D*. *starmachi* clusters within. So far only two species from this group, *D*. *cinerella* and *D*. *tonsa* Haliday, 1856, were found in the Alps ([46] see Table 1 in their study). Taking this into account and based on the phylogenetic position of *Diamesa starmachi* in the tree, we assume the misidentification of the *D*. *starmachi* labelled sequences and a false signal of paraphyly inside the *Diamesa cinerella* group. Therefore, we hypothesise that the three affected sequences are not only *D*. *starmachi* but also *D*. *cinerella* or *D*. *tonsa*, leading to the monophyly of the *Diamesa cinerella* group. This hypothesis is also supported by the known distribution of *D*. *starmachi*, which occurs in the Carpathians in mountain and low mountain streams below 1200 m a.s.l. [54, 55], therefore its occurrence in the Rotmoosache is very unlikely. However, the paraphylie of *D*. *cinerella* or *D*. *tonsa* due to potential gene flow between these two species, as supposed by Montagna *et al*. [22], cannot be excluded. The previously performed molecular studies by Montagna *et al*. [22] and Lencioni *et al*. [46] also showed that these two species (*D*. *cinerella* and *D*. *tonsa*) cluster closely intertwined. It is hypothesised that they might represent different populations of the same species [46]. The morphological identification of larvae of these two species, entirely based on the head’s colour pattern, is quite difficult or impossible and is prone to misidentification. This is stressing again the importance of collaboration between taxonomists and molecular biologists, as well as the choice of suitable marker genes. In our study, we were not able to differentiate between the larvae of these two species either. Despite the usefulness of DNA barcoding in identifying *Diamesa* larvae at the species level, there are still limitations to this approach. For example, the success of DNA barcoding relies on the availability of reference sequences for comparison. If there are no reference sequences available for a particular species, it can be difficult to identify that species using DNA barcoding. So far, our study is the first to use the ITS marker gene in chironomids, making a comparison with data resulting from other studies challenging. However, it is noticeable that our tree shows two separate main clades in the *Diamesa cinerella* group (Fig 2). Further studies, maybe including adult males, would be necessary to see if these two clades represent *D*. *cinerella* and *D*. *tonsa* as two species, or if they belong to the same species as supposed by [46].

Another interesting aspect is the phylogenetic position of *D*. *steinboecki*. In our phylogenetic tree *D*. *steinboecki*, a clade including all remaining sequences of *Diamesa* and Orthocladiinae cluster polyphyletic. However, we do not doubt the monophyly of the genus *Diamesa*, due to this seeming polyphyly. Based on morphological characteristics, *D*. *steinboecki* was supposed to feature the ancestral type of this genus [56] and is shown to have an early branching position in phylogenetic trees (see [46]). We hypothesise this position is also supported by our dataset; however, it’s just not resolved by our tree (Fig 2). Regardless of its general position in the tree, this clade forms a monophyly and can be, supported by the morphological data, clearly identified as *D*. *steinboecki*. Also, the morphological and molecular determination of *D*. *bertrami* in our tree is distinct (Fig 2).

In summary, while the ITS region can be a valuable marker for DNA barcoding, using a combination of both COI and ITS markers is often preferred to leverage their respective strengths and overcome their individual limitations. This approach ensures a more robust and reliable identification of species in diverse biological studies. Our study shows that DNA barcoding combining COI and ITS data can improve the morphological identification of *Diamesa* species in alpine streams.

As mentioned in the introduction, these harsh environments often host only a few well-adapted species [57, 58] that can withstand these conditions. Some of the *Diamesa* species seem to be obligate inhabitants of glacier-fed streams; however, other *Diamesa* species may inhabit a wider range of running waters, including lowland streams and rivers (e.g., [2, 55, 59, 60). We observed *D*. *steinboecki*, *D*. *modesta*, and *D*. *bertrami* only in the glacial-fed Rotmoosache stream (see Table 2), which is in agreement with the reported higher affinity of those species for a given habitat type than in the case of *D*. *latitarsis*, *D*. *goetghebueri*, and members of the *D*. *cinerella* group (e.g., [2]). We did not record any representatives of the *Diamesa* genus at the sampling site KT1. It is plausible that this site is a periodic stream that dries up in late summer and freezes in winter and thus does not provide a suitable habitat for *Diamesa* species.

In general, larvae of most *Diamesa* species dwell in cold water of glacial or high mountain streams. Thus, the effect of glacial retreat on *Diamesa* species will be the isolation of their populations and the potential extinction of the most cold-restricted species due to constrained habitat preferences and limited migration abilities [52]. Even though difficulties in species identification of *Diamesa* larvae often hamper detailed knowledge of their ecology, it is obvious that some species are more cold-restricted and thus more prone to extinction than others. In the Southern Alps, for example, *Diamesa steinboecki*, D. *latitarsis*, *D*. *modesta*, *D*. *goetghebueri* and *D*. *laticauda* are known to be restricted to glacial habitats [15] and thus exposed to higher risks of extinction due to the progressive shrinkage of their habitats. In addition, rare species with restricted distributions can be heavily impacted by the glacier retreat and warming water. In the Tatra Mountains, where there are no recent glaciers, populations of some *Diamesa* species (*D*. *steinboecki*, *D*. *latitarsis*, *D*. *nowickiana*) survive in a few extremely cold streams and are considered critically endangered [61].

The advances in innovative identification methods using molecular markers may overcome the issues of morphological identification and increase the knowledge of the ecology and environmental limitations of different *Diamesa* species.

Future research should also aim to apply barcoding to other chironomid life stages, such as pupae. Although pupae (and pupal exuviae) provide more reliable diagnostic features than larvae, their identification is still uncertain, and the position of some species is tentative [19].

## Conclusion

The use of DNA barcoding for species identification has numerous potential applications in ecology and conservation biology, including the monitoring of biodiversity, identification of invasive species, and assessment of the impacts of climate change on ecosystems. In the case of *Diamesa* larvae, which are important bioindicators in alpine streams, the combination of COI and ITS markers enabled a higher resolution in species identification compared to using either marker alone. This approach, supported by the utilization of morphological characters in larvae, allows for more precise species identification and the construction of distance-based phylogenetic trees for the genus *Diamesa*. Moreover, this more detailed identification within the genus can offer significant insights into the ecology of alpine streams, comprehension of which is becoming increasingly crucial in the context of the current climate changes.

## Supporting information

S1 Table Physico-chemical water parameters of studied sampling sites. (PDF)

S2 Table GenBank accession numbers of analysed sequences. GenBank database accession numbers of analysed sequences of cytochrome oxidase subunit 1 gene (COI) and ribosomal RNA gene including internal transcribed spacer 1 and 2 (ITS).
(PDF)

S3 Table GenBank accession numbers of known sequences. GenBank database accession numbers of used sequences of cytochrome oxidase subunit 1 gene (COI) and ribosomal RNA gene including internal transcribed spacer 1 and 2 (ITS).
(PDF)

S1 Fig COI phylogenetic tree. Phylogenetic tree showing species delimitation analysis based on the cytochrome oxidase subunit 1 (COI) marker gene sequences. The first three letters in each individual’s code represent the sampling site code (see Table 1).
(TIF)

S2 Fig ITS phylogenetic tree. Phylogenetic tree showing species delimitation analysis based on the internal transcribed spacer 1 and 2 (ITS) marker sequences. The first three letters in each individual’s code represent the sampling site code (see Table 1).
(TIF)
