# Supplementary material for: Molecular and morphological characterisation of larvae of the genus Diamesa Meigen, 1835 (Diptera: Chironomidae) in Alpine streams (Ötztal Alps, Austria)
Source: PLoS One. 2024 Feb 15;19(2):e0298367. doi: 10.1371/journal.pone.0298367 (PMC10868831; doi:10.1371/journal.pone.0298367)
Supplement: S1 Table — (PDF) [file pone.0298367.s001.pdf]

S1 Table. Physico-chemical water parameters of studied sampling sites.

Sampling site code	Sampling date	Temperature °C	K_{25} µS/cm	pH	Alkalinity µeq/l	Na µeq/l	K µeq/l	Ca µeq/l	Mg µeq/l	Cl µeq/l	NO_3-N µeq/l	NH_4-N µeq/l	SO_4 µeq/l	DP µeq/l	DOC mg/l	DN mg/l
**RM0**	23.08.2020	4.2	50	7.5	262	8	17	347	91.17	1.7	6	1.4	179	NA	163	110
**RM0**	21.07.2021	2.9	64	7.5	327	8	18	433	148	1.8	9	1.5	257	1.0	181	146
**RM1**	23.08.2020	6.7	64	7.6	294	8	20	415	151	3.2	5	0.6	274	NA	158	91
**RM2**	23.08.2020	7.9	72	7.6	368	9	24	473	162	7.0	5	0.7	284	NA	200	83
**RM3**	22.08.2020	10.6	66	7.6	328	10	34	428	145	2.2	4	0.8	271	NA	199	73
**RM4**	22.08.2020	12.5	71	7.7	356	10	36	455	150	1.7	4	0.7	284	NA	176	73
**RM4**	21.07.2021	10.1	128	7.7	585	16	40	836	318	2.2	8	0.7	603	1.4	268	121
**KT1**	20.07.2021	5.3	212	6.9	74	31	50	1260	696	3.8	9	0.1	1839	0.4	208	130
**KT2**	20.07.2021	8.6	154	7.1	82	28	34	794	487	4.0	7	0.1	1248	0.1	177	103
**TJ1**	19.07.2021	6.7	126	6.9	70	25	29	577	459	3.9	10	0.1	1007	0.8	123	137
