# Supplementary material for: Molecular and morphological characterisation of larvae of the genus Diamesa Meigen, 1835 (Diptera: Chironomidae) in Alpine streams (Ötztal Alps, Austria)
Source: PLoS One. 2024 Feb 15;19(2):e0298367. doi: 10.1371/journal.pone.0298367 (PMC10868831; doi:10.1371/journal.pone.0298367)
Supplement: S2 Table — GenBank database accession numbers of analysed sequences of cytochrome oxidase subunit 1 gene (COI) and ribosomal RNA gene including internal transcribed spacer 1 and 2 (ITS). (PDF) [file pone.0298367.s002.pdf]

S2 Table. GenBank accession numbers of analysed sequences. GenBank database accession numbers of analysed sequences of cytochrome oxidase subunit 1 gene (COI) and ribosomal RNA gene including internal transcribed spacer 1 and 2 (ITS).

Sample id	Determined species	NCBI accession number for analysed sequence of	
		COI	ITS
RM0-001	*Diamesa modesta*	OR500832	OR502728
RM0-002	*Diamesa modesta*	OR500833	OR502729
RM0-005	*Diamesa steinboecki*	OR500866	OR502762
RM0-006	*Diamesa steinboecki*	OR500867	OR502763
RM0-007	*Diamesa modesta*	OR500834	OR502730
RM0-008	*Diamesa modesta*	OR500835	OR502731
RM0-009	*Diamesa modesta*	OR500836	OR502732
RM0-010	*Diamesa modesta*	OR500837	OR502733
RM0-011	*Diamesa modesta*	OR500838	OR502734
RM0-012	*Diamesa steinboecki*	OR500868	OR502764
RM0-013	*Diamesa modesta*	OR500839	OR502735
RM0-014	*Diamesa bertrami*	OR500783	OR502679
RM0-015	*Diamesa steinboecki*	OR500869	OR502765
RM0-016	*Diamesa steinboecki*	OR500870	OR502766
RM0-017	*Diamesa modesta*	OR500840	OR502736
RM0-018	*Diamesa modesta*	OR500841	OR502737
RM0-019	*Diamesa modesta*	OR500842	OR502738
RM0-020	*Diamesa goetghebueri*	OR500807	OR502703
RM0-021	*Diamesa modesta*	OR500843	OR502739
RM0-022	*Diamesa steinboecki*	OR500871	OR502767
RM0-023	*Diamesa bertrami*	OR500784	OR502680
RM0-024	*Diamesa cinerella* gr.	OR500793	OR502689
RM0-025	*Diamesa cinerella* gr.	OR500794	OR502690
RM0-026	*Diamesa steinboecki*	OR500872	OR502768
RM0-027	*Diamesa steinboecki*	OR500873	OR502769
RM0-028	*Diamesa cinerella* gr.	OR500795	OR502691
RM0-029	*Diamesa latitarsis*	OR500818	OR502714
RM0-030	*Diamesa steinboecki*	OR500874	OR502770
RM0-031	*Diamesa steinboecki*	OR500875	OR502771
RM0-032	*Diamesa steinboecki*	OR500876	OR502772

RM0-033	*Diamesa cinerella* gr.	OR500796	OR502692
RM0-034	*Diamesa cinerella* gr.	OR500797	OR502693
RM0-035	*Diamesa steinboecki*	OR500877	OR502773
RM0-036	*Diamesa bertrami*	OR500785	OR502681
RM0-037	*Diamesa goetghebueri*	OR500808	OR502704
RM0-100	*Diamesa steinboecki*	OR500878	OR502774
RM0-101	*Diamesa modesta*	OR500844	OR502740
RM0-102	*Diamesa steinboecki*	OR500879	OR502775
RM0-103	*Diamesa steinboecki*	OR500880	OR502776
RM0-104	*Diamesa steinboecki*	OR500881	OR502777
RM1-001	*Diamesa modesta*	OR500845	OR502741
RM1-002	*Diamesa modesta*	OR500846	OR502742
RM1-003	*Diamesa modesta*	OR500847	OR502743
RM1-004	*Diamesa modesta*	OR500848	OR502744
RM1-005	*Diamesa modesta*	OR500849	OR502745
RM1-006	*Diamesa steinboecki*	OR500882	OR502778
RM1-007	*Diamesa modesta*	OR500850	OR502746
RM1-008	*Diamesa modesta*	OR500851	OR502747
RM1-009	*Diamesa goetghebueri*	OR500809	OR502705
RM1-010	*Diamesa modesta*	OR500852	OR502748
RM1-012	*Diamesa modesta*	OR500853	OR502749
RM2-001	*Diamesa modesta*	OR500854	OR502750
RM2-002	*Diamesa bertrami*	OR500786	OR502682
RM2-003	*Diamesa modesta*	OR500855	OR502751
RM2-004	*Diamesa modesta*	OR500856	OR502752
RM2-005	*Diamesa modesta*	OR500857	OR502753
RM2-006	*Diamesa goetghebueri*	OR500810	OR502706
RM2-008	*Diamesa modesta*	OR500858	OR502754
RM2-009	*Diamesa steinboecki*	OR500883	OR502779
RM2-010	*Diamesa modesta*	OR500859	OR502755
RM3-001	*Diamesa modesta*	OR500860	OR502756
RM3-002	*Diamesa modesta*	OR500861	OR502757
RM3-003	*Diamesa bertrami*	OR500787	OR502683
RM3-004	*Diamesa latitarsis* gr.	OR500829	OR502725
RM3-005	*Diamesa bertrami*	OR500788	OR502684
RM3-006	*Diamesa modesta*	OR500862	OR502758
RM3-007	*Diamesa goetghebueri*	OR500811	OR502707

RM3-008	*Diamesa latitarsis*	OR500819	OR502715
RM3-009	*Diamesa latitarsis* gr.	OR500830	OR502726
RM3-010	*Diamesa modesta*	OR500863	OR502759
RM3-011	*Pseudodiamesa branickii*	OR501473	OR502782
RM3-012	*Pseudodiamesa branickii*	OR501474	OR502783
RM3-013	*Diamesa bertrami*	OR500789	OR502685
RM4-002	*Diamesa goetghebueri*	OR500812	OR502708
RM4-003	*Diamesa modesta*	OR500864	OR502760
RM4-004	*Diamesa bertrami*	OR500790	OR502686
RM4-005	*Diamesa bertrami*	OR500791	OR502687
RM4-006	*Pseudokiefferiella parva*	OR501476	OR502785
RM4-007	*Pseudodiamesa branickii*	OR501475	OR502784
RM4-009	*Diamesa cinerella* gr.	OR500798	OR502694
RM4-011	*Diamesa bertrami*	OR500792	OR502688
RM4-012	*Diamesa modesta*	OR500865	OR502761
RM4-013	*Diamesa steinboecki*	OR500884	OR502780
RM4-014	*Diamesa steinboecki*	OR500885	OR502781
RM4-015	*Diamesa goetghebueri*	OR500813	OR502709
RM4-016	*Diamesa latitarsis*	OR500820	OR502716
RM4-017	*Diamesa cinerella* gr.	OR500799	OR502695
RM4-018	*Diamesa latitarsis*	OR500821	OR502717
RM4-019	*Diamesa cinerella* gr.	OR500800	OR502696
RM4-020	*Diamesa cinerella* gr.	OR500801	OR502697
RM4-021	*Diamesa latitarsis*	OR500822	OR502718
RM4-022	*Diamesa cinerella* gr.	OR500802	OR502698
RM4-023	*Diamesa latitarsis*	OR500823	OR502719
RM4-024	*Diamesa latitarsis*	OR500824	OR502720
RM4-025	*Diamesa goetghebueri*	OR500814	OR502710
RM4-026	*Diamesa goetghebueri*	OR500815	OR502711
RM4-027	*Diamesa cinerella* gr.	OR500803	OR502699
RM4-028	*Diamesa cinerella* gr.	OR500804	OR502700
RM4-029	*Diamesa cinerella* gr.	OR500805	OR502701
KT1-005	*Pseudokiefferiella parva*	OR501477	OR502786
KT1-006	*Pseudokiefferiella parva*	OR501478	OR502787
KT1-007	*Pseudokiefferiella parva*	OR501479	OR502788
KT1-011	*Pseudokiefferiella parva*	OR501480	OR502789
KT2-001	*Diamesa latitarsis*	OR500825	OR502721

KT2-002	*Diamesa cinerella* gr.	OR500806	OR502702
KT2-003	*Diamesa goetghebueri*	OR500816	OR502712
KT2-004	*Pseudokiefferiella parva*	OR501481	OR502790
KT2-006	*Pseudokiefferiella parva*	OR501483	OR502791
KT2-007	*Diamesa latitarsis*	OR500826	OR502722
KT2-008	*Diamesa latitarsis*	OR500827	OR502723
KT2-009	*Diamesa latitarsis*	OR500828	OR502724
KT2-014	*Pseudokiefferiella parva*	OR501485	OR502792
TJ1-003	*Pseudokiefferiella parva*	OR501486	OR502793
TJ1-004	*Pseudokiefferiella parva*	OR501487	OR502794
TJ1-005	*Diamesa goetghebueri*	OR500817	OR502713
TJ1-006	*Diamesa latitarsis* gr.	OR500831	OR502727
TJ1-007	*Pseudokiefferiella parva*	OR501488	OR502795
TJ1-008	*Pseudokiefferiella parva*	OR501489	OR502796
TJ1-009	*Pseudokiefferiella parva*	OR501490	OR502797

4
