# Supplementary material for: Molecular and morphological characterisation of larvae of the genus Diamesa Meigen, 1835 (Diptera: Chironomidae) in Alpine streams (Ötztal Alps, Austria)
Source: PLoS One. 2024 Feb 15;19(2):e0298367. doi: 10.1371/journal.pone.0298367 (PMC10868831; doi:10.1371/journal.pone.0298367)
Supplement: S3 Table — GenBank database accession numbers of used sequences of cytochrome oxidase subunit 1 gene (COI) and ribosomal RNA gene including internal transcribed spacer 1 and 2 (ITS). (PDF) [file pone.0298367.s003.pdf]

S3 Table. GenBank accession numbers of known sequences. GenBank database accession numbers of used sequences of cytochrome oxidase subunit 1 gene (COI) and ribosomal RNA gene including internal transcribed spacer 1 and 2 (ITS).

Sample id	Species	NCBI accession number for analysed sequence of	
		COI	ITS
outgroup	*Culex pipiens*	MT519668.1	MT808425.1
NCBI_*D.latitarsis*	*Diamesa latitarsis*	LN897666.1	NA
NCBI_*D.bertrami*	*Diamesa bertrami*	MT048138.1	NA
NCBI_*D.steinboecki*	*Diamesa steinboecki*	LN897628.1	NA
NCBI_*D.cinerella*	*Diamesa cinerella*	LN897682.1	NA
NCBI_*D.goetghebueri*	*Diamesa goetghebueri*	LN897607.1	NA
NCBI_*D.modesta*	*Diamesa modesta*	LN897649.1	NA
NCBI_*P.parva*	*Pseudokiefferiella parva*	MZ659124.1	NA
NCBI_*R.effusus*	*Rheocricotopus effusus*	MZ660336.1	NA
NCBI_*B.bifida*	*Brillia bifida*	MZ660470.1	NA
NCBI_*D.zernyi*	*Diamesa zernyi*	LN897612.1	NA
NCBI_*D.tonsa*	*Diamesa tonsa*	LN897614.1	NA
NCBI_*O.frigidus*	*Orthocladius frigidus*	MZ660393.1	NA
NCBI_*P.branickii*	*Pseudodiamesa branickii*	MZ656789.1	NA
NCBI_*D.lindrothi*	*Diamesa lindrothi*	OQ282685.1	NA
NCBI_*D.martae*	*Diamesa martae*	OQ282653.1	NA
