# Supplementary figures and images for: Molecular and morphological characterisation of larvae of the genus Diamesa Meigen, 1835 (Diptera: Chironomidae) in Alpine streams (Ötztal Alps, Austria)
Source: PLoS One. 2024 Feb 15;19(2):e0298367. doi: 10.1371/journal.pone.0298367 (PMC10868831; doi:10.1371/journal.pone.0298367)

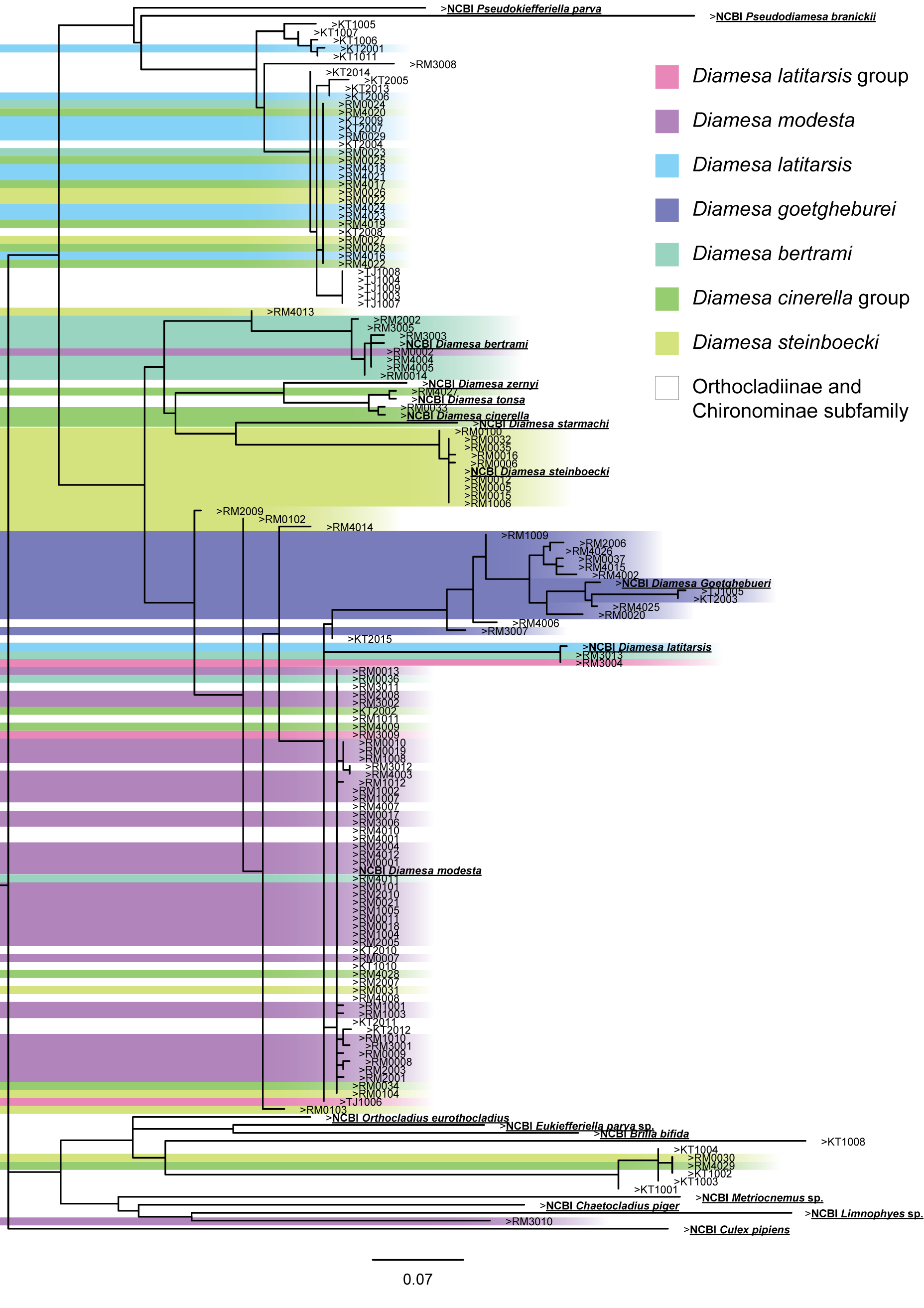

Supplement: S1 Fig — Phylogenetic tree showing species delimitation analysis based on the cytochrome oxidase subunit 1 (COI) marker gene sequences. The first three letters in each individual’s code represent the sampling site code (see Table 1). (TIF) [file pone.0298367.s004.tif]

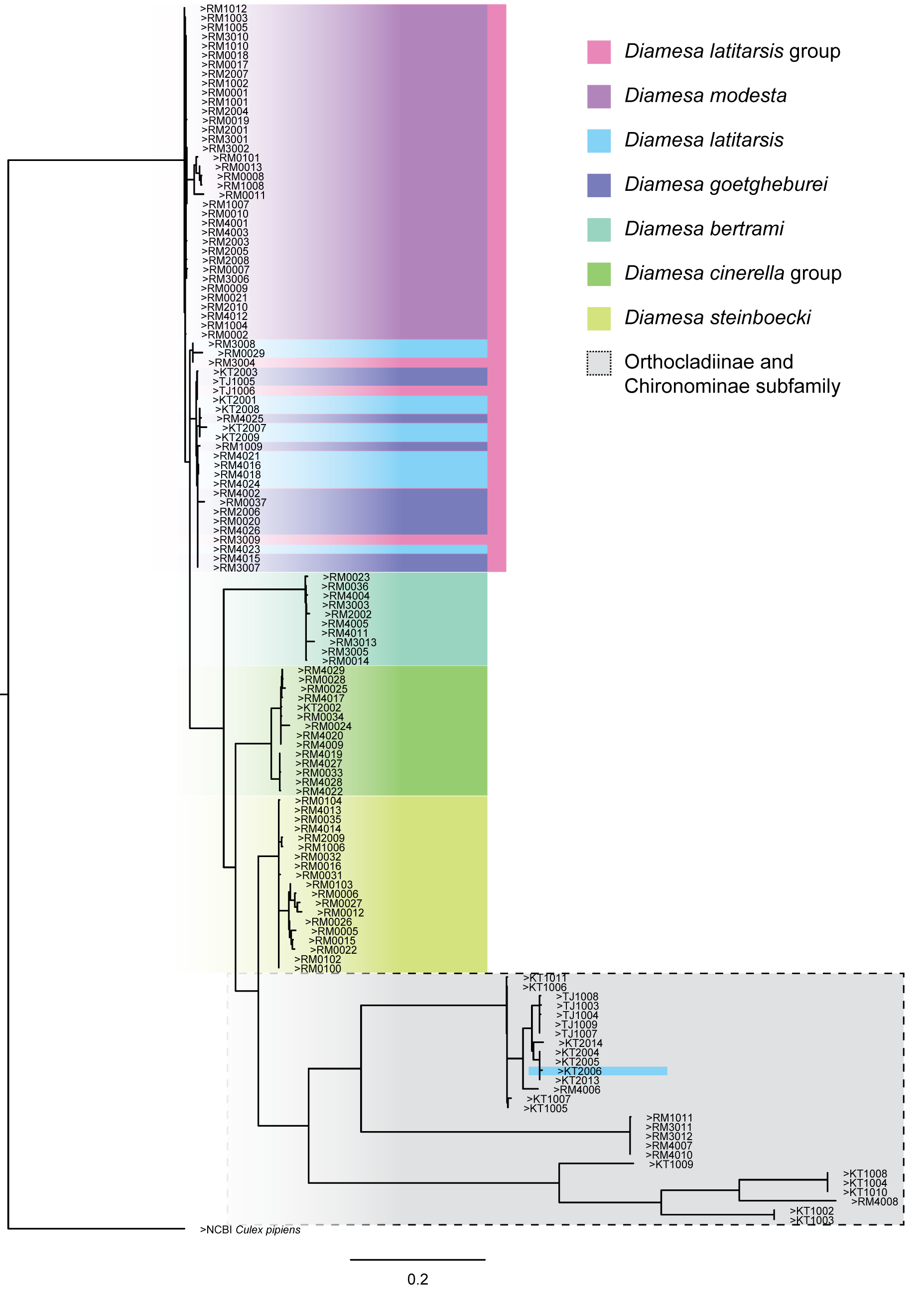

Supplement: S2 Fig — Phylogenetic tree showing species delimitation analysis based on the internal transcribed spacer 1 and 2 (ITS) marker sequences. The first three letters in each individual’s code represent the sampling site code (see Table 1). (TIF) [file pone.0298367.s005.tif]
